# A protocol for retrospective evaluation of potential patient-level harms of an AI tool for blood culture stewardship

**DOI:** 10.1017/ash.2026.10358

**Published:** 2026-04-07

**Authors:** Michiel Schinkel, Irene Reijers, Anuschka van der Zaag, Sheena Bhagirath, Lotte Lintmeijer, Liesbeth bij de Vaate, Hazra Moeniralam, Prabath Nanayakkara, Erik Schaftenaar, Hanneke Boon

**Affiliations:** 1 Amsterdam UMC - Locatie VUMC: Amsterdam UMC Locatie VUmc, Netherlands; 2 Sint Antonius Ziekenhuis, Netherlands

## Abstract

**Background:**

Artificial intelligence (AI)-based clinical decision support tools are increasingly developed for diagnostic stewardship, yet their clinical adoption remains limited. A barrier to implementation is concern about potential patient-level harm, particularly when algorithms recommend withholding diagnostic tests that would otherwise be standard practice. Existing evaluation frameworks emphasize aggregate performance metrics but do not provide structured methods to assess clinical consequences of algorithmic errors prior to implementation.

**Objective:**

Developing a protocol for retrospective, patient-level evaluation of potential harms associated with false-negative predictions of an AI-tool for blood culture stewardship in the emergency department.

**Methods:**

We developed a protocol to identify and evaluate cases at potential risk of harm following retrospective application of an AI-model predicting blood culture outcomes. False-negative cases, defined as positive blood cultures with a model-predicted probability below 5%, are selected after exclusion of contaminants and clinical scenarios in which the algorithm would not be applied. Multidisciplinary experts perform case-by-case evaluation using a questionnaire covering three domains: antibiotic management, diagnostic procedures, and patient outcomes, supplemented by overall harm and cost assessments. Inter-observer agreement is quantified, and discrepancies are resolved through expert adjudication.

**Expected outcomes and significance:**

This protocol is designed as a preimplementation safety assessment to support go/no-go decisions for advancing AI tools into clinical research or practice. By operationalizing patient-level harm assessment using routinely collected data, the framework complements existing AI evaluation standards and addresses a critical gap in diagnostic stewardship. Although developed for blood culture stewardship, the protocol may be adaptable to other AI-based decision support tools in infectious diseases and beyond.

## Introduction

In healthcare research, a vast number of artificial intelligence (AI) algorithms have been developed, with over 114,000 studies on clinical AI models published to date.^1^ However, adoption, integration, and deployment of AI into the clinical workflow have proven to be a true challenge, with fewer than 1% of developed AI models reaching routine clinical use.^1^ Perceived or real barriers for implementation are numerous. Beyond financial constraints, technical challenges, data bias, and regulatory uncertainty, concern about potential patient-level harm remains a key obstacle to clinical adoption.^2,3^ This is particularly true in cases where algorithms recommend withholding diagnostic tests or therapeutic interventions that would otherwise constitute standard practice.

Methodologically rigorous evaluation is essential to generate trustworthy evidence on the safety and performance of clinical AI prior to implementation, particularly when algorithms recommend withholding diagnostic tests or treatments. Assessing potential harm from such decisions is challenging using high-level observational data, as counterfactual outcomes are inherently unobservable and false-negative predictions may have important clinical consequences. At the same time, traditional causal inference approaches, including randomized controlled trials, are often impractical or infeasible given the speed, scale, and diversity of AI innovations entering healthcare.^4^ To our knowledge, no published protocols specifically address this gap for clinical AI tools, despite their potential to provide valuable insights into algorithmic failure modes and patient safety prior to clinical use.

Here, we propose a protocol for in-depth, retrospective analysis of cases where an already developed AI tool for blood culture stewardship has made a false-negative prediction that may thus put a patient at risk of potential harm. The protocol is intended to complement existing standards for AI development and serve as a preimplementation safety evaluation before using the blood culture tool in our hospital. The resulting insights into direction and severity of possible harm to patients may serve as the basis for the final decision to start using a tool in a research or clinical setting.

## Methods and analysis

### Algorithm background: development

As a use case to develop this protocol, we consider our extreme gradient-boosting decision tree (XGBoost) algorithm that predicts the results of blood cultures in the emergency department (ED). We first developed the model at the Amsterdam University Medical Center (AUMC) in the Netherlands.^5,6^ Its development, including the rationale for feature selection, extraction of data, patient exclusion criteria, handling of missing data, target development as well as its final performance characteristics in earlier external validation sets has been described extensively elsewhere.^5,6^ To complement the initial external validation and aid the implementation process in other hospitals, a large-scale external validation will be performed on data from electronic health records (EHR) of all adult patients visiting the ED of the St. Antonius Hospital between January 1^t^, 2018, and July 1, 2023. The St. Antonius Hospital is a 500-bed teaching hospital in the Netherlands. On this data set, an in-depth clinical evaluation of potential harms of (retrospective) application of the algorithm will be performed according to the protocol described below.

### Inclusion of data

**Figure 1.**
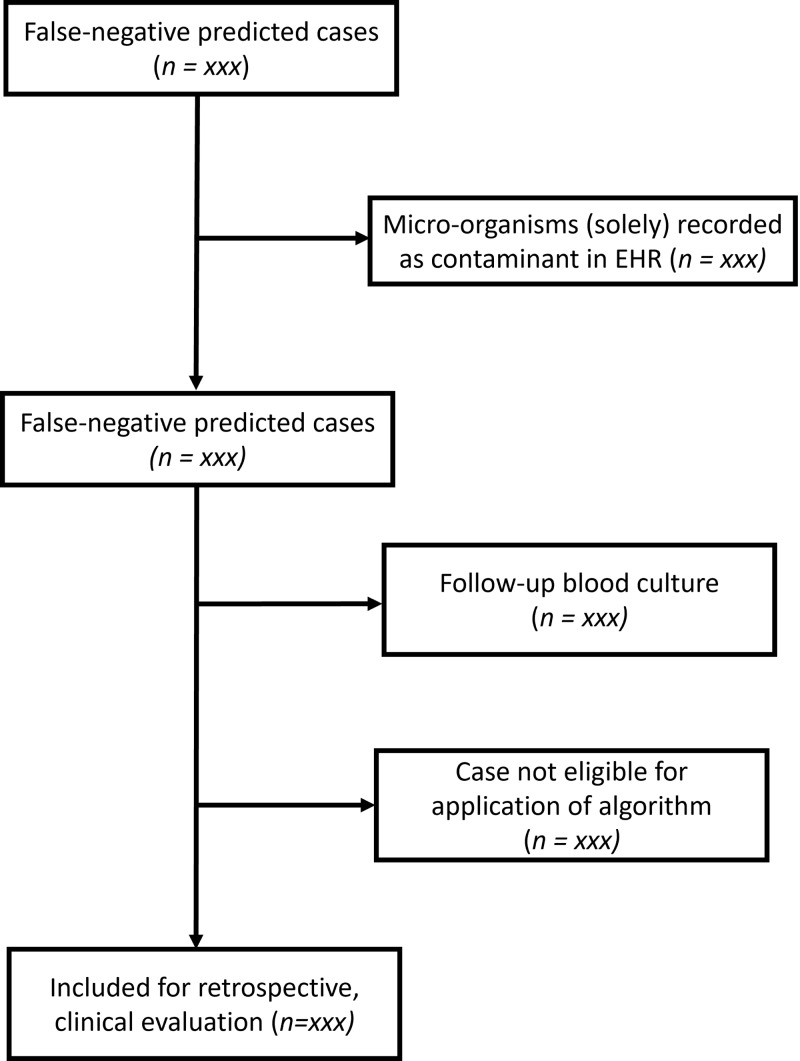
Flowchart to evaluate eligibility of the case for in-depth retrospective clinical evaluation.

A graphic representation of the inclusion and categorization of cases is depicted in Figure 1. The first step in our proposed protocol is to identify the cases that are at potential risk of harm by retrospectively applying the algorithm to the described data set (Figure 1). Data will be extracted from the Electronic Health Record system (EHR, EPIC, Epic Systems Corporation). We then select cases in which a blood culture yielded a positive result, while the model predicted a probability of positivity below 5% and would therefore recommend not obtaining a culture. This constitutes a false-negative prediction. A probability of less than .05 (5%) was chosen in consensus during the development phase of this algorithm, as higher probabilities of missing a positive blood culture were regarded as clinically unacceptable. After the initial selection, an additional screening is performed to filter out blood cultures which are marked as contaminants in the EHR, which constitute false-positives and should not be considered. Those cultures will be recategorized as true negative. In a final step, a manual check is performed to the remaining cases at potential harm to filter out those in whom the algorithm would not be used in practice (eg, patients with neutropenia, suspected endocarditis, or follow-up cultures in patients with previously positive cultures). Those cultures will be recategorized not eligible for inclusion.” An overview of the counts in the various groups of inclusion and exclusion will be provided, as well as aggregate data on characteristics such as culture positivity rates in those groups. These data specifically may be interesting to estimate case-mix differences between the development setting and our hospital.

### Study design: clinical evaluation

The main part of the protocol aims to help identify the possible clinical consequences of false-negative predicted blood cultures in detail through a case-by-case evaluation (Table 1). Through panel discussions, experts in the fields of microbiology, infectious diseases, internal medicine, intensive care, and epidemiology reached consensus on nine questions in three domains to cover the potential (detrimental) consequences of a false-negative blood culture prediction for the individual patient. The domains in which harm might occur because of a false-negative prediction were identified to lie within the areas of antibiotics prescription, diagnostics, and patient outcomes. The panel discussions, though unstructured, were deliberately focused on outcomes that are also being evaluated in a prospective randomized controlled trial using the same algorithm, to ensure alignment of domains for future comparison.^7^ With respect to antibiotics, inappropriate choice of empirical antibiotics or of narrow-spectrum antibiotics (Q1 and Q2, respectively), and insufficient length of antibiotics therapy (Q3) were deemed to be of greatest concern. Within the area of diagnostics, the greatest concern was that unnecessary (invasive) diagnostics would be ordered, causing discomfort for the patient (Q4). Concerns about missing the need for admission, unnecessary additional days in the hospital or worse-than-expected outcomes, were captured in the final three questions (Q5–7). As the probability exists that the patient may still experience harm, despite zero or only minor alterations in therapeutics, diagnostics, or outcomes, two final questions (Q8–9) on general harm and costs were added. For the financial category, the reviewers will be instructed to consider potential biases such as survivorship in their judgment.

**Table 1.** Domains and subdomains of interest for clinical evaluation on case-by-case basis

Domain	Subdomain	Question	Score
**Antibiotics**	Empirical therapy	Would an FN prediction have negatively affected choice of empirical therapy?	□ Very unlikely □ Possible □ Very likely
	Narrow-spectrum therapy	Would an FN prediction have negatively affected choice of narrow-spectrum therapy?	□ Very unlikely □ Possible □ Very likely
	Length of therapy	Would an FN prediction have negatively affected choice of length of therapy?	□ Very unlikely □ Possible □ Very likely
**Diagnostics**	Additional diagnostic procedures	Could an FN prediction have led to unnecessary diagnostic procedures?	□ Very unlikely □ Possible □ Very likely
**Outcome**	Admission	Could an FN prediction have negatively affected the decision for admission or readmission?	□ Very unlikely □ Possible □ Very likely
	Length of stay	Could an FN prediction have negatively affected length of hospital stay?	□ Very unlikely □ Possible □ Very likely
	Morbidity	Could an FN prediction have led to worse-than-expected morbidity (including mortality)?	□ Very unlikely □ Possible □ Very likely
**General**		Would an FN prediction, overall, have resulted in disproportionate harm to the patient?	□ Very unlikely □ Possible □ Very likely
**Financial**		Would an FN prediction have led to increased hospital costs?	□ Very unlikely □ Possible □ Very likely

FN, false-negative.

Questions will be scored on a 3-point forced-choice Likert scale without a neutral midpoint. The absence of a neutral midpoint was deliberate, to force the reviewing expert to make a definitive judgment on the case, rather than defaulting to an undecided position. A 3-point scale is thought to simplify the decision process and therefore appeal to time-constrained experts having to judge a high number of cases. Per question, exemplary cases were constructed as a reference to help the medical expert judge the severity of the case and correctly interpret the question. Before consolidation of the questionnaire, two experts each assessed ten cases to judge the correct interpretability of the questions.

### Statistical analysis

Two observers (MS and IM) will individually score all false-negative predicted cases. First, each case will be screened to confirm that the case should not be recategorized as “true negative or is not eligible for inclusion. Scores per question will be expressed on a 3-point forced-choice Likert scale without a neutral midpoint and expressed as percentages. Overall scores per question will be calculated per reviewer as well as for both reviewers combined and expressed as percentages.

To identify any possible differences between observers’ ratings, a linearly weighted Cohen’s kappa as well as percent agreement will be calculated for individual scores between the two observers. Krippendorff’s alpha will be calculated for the full questionnaire (Q1–7). As the final questions on general harm and costs are related to all other questions, these will not be included in the Krippendorff alpha calculation. The analyses will be performed in Python (version 3.7 +).

To come to a final consensus on clinical impact of a false-negative prediction per case, small discrepancies between the observers’ ratings will be resolved among the two initial observers. Large, or unresolvable discrepancies in a case, that is more than 2 questions scored on opposite ends of the Likert scale, will be resolved by an expert panel of two independent medical specialists to come to a consensus on a final adjudication. Through these steps, a final consensus on all cases is necessary to present the results.

### Ethical considerations

Both the AUMC and St. Antonius Hospital institutional review boards waived the review of this study as the Medical Research Involving Human Subjects Act did not apply (IRB number: IRB00002991; case: 2020.486). No data will be extracted for patients who have registered that their data should not be used in scientific research. Publication of results of this study will adhere to the Transparent reporting of a multivariable prediction model for individual prognosis or diagnosis (TRIPOD + AI) reporting guidelines.

## Discussion

The protocol described here is primarily designed to structurally evaluate the magnitude and severity of potential harm to patients following application of our blood culture algorithm and will serve as the basis for the decision to implement the tool in our clinical practice.

Existing reporting guidelines and evaluation frameworks, such as TRIPOD + AI, APPRAISE-AI, STARD-AI, and DECIDE-AI, provide important guidance on AI model development, validation, and testing.^8–11^ However, they are not designed to assess the patient-level clinical consequences of algorithmic errors. We consider this a substantial gap in current AI evaluation and implementation, particularly for tools intended to support diagnostic or therapeutic decision making. A recent study by Fabre et al confirms that concerns for missing infections and associated patient harms are major barriers to blood culture stewardship.^12^ As our blood culture AI tool transitions toward use in a clinical research setting, this gap presents a key challenge. Therefore, we developed the here proposed protocol, which is best positioned as a preimplementation safety assessment, complementing existing guidelines. It is not intended to replace randomized trials, when these are feasible or required, but rather to support a go/no-go decision on whether an AI tool is sufficiently safe to advance into clinical research or routine practice. By systematically examining cases at high risk of patient-level harms of algorithmic errors, in this use case through false-negative predictions, it addresses the specific risks that most strongly influence safety, clinician trust, and adoption. Furthermore, when safety concerns do arise, the thorough evaluation of these cases may help identify characteristics of patients who are at higher risk of false-negative predictions and possibly help refine the model or the selection of patients to which it should or should not be applied.

Although the current protocol was developed in the context of a diagnostic stewardship tool for blood cultures, we believe it is applicable to a broad range of AI-based tools in infectious diseases and clinical microbiology. With minimal adaptation of the questions and domains, the framework can be reused by other researchers or healthcare institutions, as it targets outcome categories that are common across these fields. Interestingly, recent work by Rodriquez-Nava et al shows that Large Language Models (LLM) may be increasingly capable of recognizing inappropriate blood cultures.^13^ Our protocol could help objectify potential harms of such LLMs, which are increasingly being developed and implemented. By focusing on scenarios most likely to undermine clinician trust, the protocol addresses a central barrier to the implementation of AI-based clinical decision support. Importantly, the approach is resource-efficient and can be applied using routinely collected clinical data, even in settings without the infrastructure for large prospective trials. In our use case, for example, the algorithm has previously shown >95% sensitivity in a population with around 11% true positive blood cultures. Expected workload for evaluation thus amounts to approximately .6% of available cases. This targeted strategy enables in-depth assessment of potential harms while maintaining scalability.^13^

Several limitations to our protocol should be acknowledged. Firstly, the protocol relies on retrospective observational data, and no formal causal relationship can be established between withholding the blood culture and possible negative outcomes. Despite the high volume of tests, in EDs, the possibility remains that consistent false-negative predictions for rare conditions may go unnoticed. Secondly, this protocol is intentionally designed to generate expert-based clinical evaluation of possible health outcomes. Clinicians from different areas of expertise, settings, or health care systems may thus come to different clinical evaluations. However, only large, randomized controlled trials can generate measurable outcomes and a definitive assessment of harm. Finally, this framework intentionally focuses on harms related to omitted diagnostics because of AI suggestions and does not assess potential harms from overdiagnosis or overtreatment. The primary aim is to address concerns about missed diagnoses among potential users, which is a major barrier to implementation in this particular use case. Yet, an opposite approach of assessing potentially beneficial effects of using the AI algorithm should be just as viable, if that is the specific barrier for a certain tool.

## Conclusion

As AI tools increasingly influence diagnostic and therapeutic decisions, evaluation strategies must be tailored to specific requirements and barriers to implementation. The protocol presented here offers a practical, reproducible method to assess cases at high risk of potential harm by AI tools, using in-depth reviews with routinely available data. By explicitly focusing on scenarios that challenge clinician trust and adoption, this framework supports safer and more transparent implementation of AI in diagnostic stewardship. Broader adoption of such structured risk assessments may help inform clinical deliberation and support more transparent implementation of clinical AI tools in line with core medical principles.
